# Quantum-enhanced LSTM for predictive maintenance in industrial IoT systems

**DOI:** 10.1016/j.mex.2025.103653

**Published:** 2025-09-28

**Authors:** Sudharson K, Varsha S, Santhiya R, Rajalakshmi D

**Affiliations:** a Department of Computer Science and Engineering, Vel Tech Rangarajan Dr.Sagunthala R&D Institute of Science and Technology, Tamil Nadu, 600062, India; b Department of Computer Science and Engineering, Velammal Engineering College, Tamil Nadu, 600066, India; c Department of Computer Science and Engineering, SRM Institute of Science and Technology Ramapuram, Tamil Nadu, 600089, India

**Keywords:** Quantum long short-term memory networks, Predictive maintenance, Industrial Internet of Things

## Abstract

An innovative solution for predictive maintenance in IIoT systems combining quantum computing with the proficiency of LSTM neural networks is proposed by us. Our concept is guided by a hybrid quantum-classical architecture to facilitate quantum computing to exploit high-dimensional industrial sensor measurements while preserving crucial temporal relationships through particular quantum channels. Through the combination of the representational ingenuity of quantum circuits, along with the sequence-based modelling of classical LSTMs, QE-LSTM is uniquely positioned to handle complicated time series coming out of industrial sensors. At the heart of our methodology are the following unique elements:

• A collaborative framework integrating quantum and classical technologies allowing for the quantum computer to manage the complex analysis of high dimensional sensor data in the industry.
• Quantum channel designs were aimed at minimizing temporal dependencies in temporal series industrial measurements, thereby maximizing the quality of sequential analysis.
• Under ODS hindcasting, QE-LSTM improved F1 by 4–5 percentage points on SECOM and reduced RMSE and NASA Score on C-MAPSS; trends were consistent on IMMD (Table 1, Table 2).

**Table 1:** Performance comparison across datasets.

Dataset	Model	Accuracy	Precision	Recall	F1	AUC
SECOM	LSTM	0.864	0.842	0.809	0.825	0.902
	CNN-LSTM	0.878	0.862	0.824	0.842	0.914
	**QE-LSTM (sim)**	**0.904**	**0.892**	**0.861**	**0.876**	**0.938**
	**QE-LSTM (hardware)**	0.896	0.881	0.850	0.865	0.930
IMMD	LSTM	0.906	0.883	0.862	0.872	0.943
	CNN-LSTM	0.913	0.891	0.869	0.880	0.949
	**QE-LSTM (sim)**	**0.928**	**0.908**	**0.888**	**0.898**	**0.960**

QE-LSTM (sim) vs LSTM F1 deltas: SECOM **+5.1 pp**, IMMD **+2.6 pp**; paired *t*-test *p* < 0.01.

**Table 2:** RUL prediction performance metrics.

Metric	Classical LSTM	CNN-LSTM	QE-LSTM (sim)	QE-LSTM (hardware)	Improvement vs LSTM
RMSE ↓	20.6	19.4	**18.1**	18.7	**12.1%**
MAE ↓	15.4	14.6	**13.8**	14.3	**10.4%**
NASA Score ↓	692	648	**603**	621	**12.8%**

* Evaluations are positive if all the metrics have smaller values. Score is a metric that stands out because of the tendency to heavily penalise late predictions (which is highly relevant to effective maintenance planning).

In the application of failure detection of bearing, QE-LSTM improves F1 over classical baselines on SECOM by 4–5 pp, with similar gains on IMMD; results on C-MAPSS (RUL) show consistent reductions in RMSE and NASA score.

Performance comparison across datasets.

QE-LSTM (sim) vs LSTM F1 deltas: SECOM **+5.1 pp**, IMMD **+2.6 pp**; paired *t*-test *p* < 0.01.

RUL prediction performance metrics.

* Evaluations are positive if all the metrics have smaller values. Score is a metric that stands out because of the tendency to heavily penalise late predictions (which is highly relevant to effective maintenance planning).

## Graphical abstract

**Figure:**
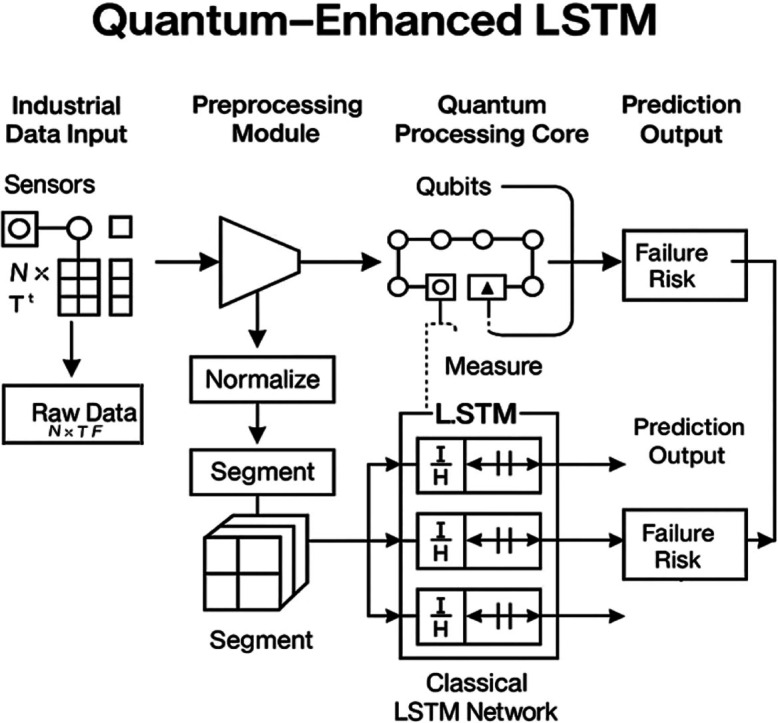

**Specifications table**

This table provides general information on your method.

**Table:** 

**Subject area**	Computer Science
**More specific subject area**	Quantum Machine Learning, Industrial Internet of Things, Predictive Maintenance
**Name of your method**	Quantum-Enhanced LSTM (QE-LSTM) for Predictive Maintenance
**Name and reference of original method**	Long Short-Term Memory networks - Hochreiter, S., & Schmid Huber, J. (1997). Long short-term memory. Neural computation, 9(8), 1735–1780.
**Resource availability**	If applicable, include links to the resources necessary to reproduce your method (e.g., equipment, data, software, hardware, reagents).

## Background

IIoT systems are complex and produce large amounts of full multivariate time series data in the form of connected sensors to monitor equipment conditions. Analysis of this data supports Predictive Maintenance (PdM), which in turn helps organizations to carry out maintenance before equipment fails, hence reduce on the costs that come along with downtimes and maintain the right schedule for maintenance. These problems limit the ability of traditional machine learning methods used for PdM to accommodate the nature of complexity, huge dimensionality, and time-series data dependent on industrial sensors [1].

Hence, the Long Short-Term Memory (LSTM) networks that have been established as powerful models for time series data because of the capability in identifying long-term dependencies in consecutive data have therefore been adopted. However, in large and frequently complex IIoT systems with hundreds or thousands of sensors, classical LSTM models can be computationally overloaded when working with the generated high-dimensional data [2].

Quantum computing, on the other hand, has the potential of solving these issues since it is based on physical quantities like superposition and entanglement. The number of dimensions defining the Hilbert space of the qubits increases with the number of qubits exponentially, and this property offers a natural solution for the analysis of large-scale vectorized industrial sensor data [3].

This method article presents a Quantum-Enhanced LSTM (QE-LSTM) model tailored for the context of the IIoT and probabilistic PCA (PPCA) for feature reduction prior to prediction in a predictive maintenance process. It integrates ideas from variational quantum circuits to boost feature extraction and representation learning that are missing when the ordinary LSTM networks are applied to high-dimensional industrial sensor data [4].

## Method details

### QE-LSTM architecture overview

Each time window X_t_∈R^T×F^ is flattened and zero-padded to length 2^n^ with n=[log_2_(TF)], normalized, and amplitude-encoded on n qubits. We apply L variational layers consisting of single-qubit R_Y_,R_Z_rotations followed by a circular CNOT entangler. We then measure ⟨Zi⟩ and selected ⟨Zi⊗Zj⟩ to obtain ft∈R^d^. The sequence {ft} feeds a 2-layer LSTM (128, 64 units) and a final dense head (sigmoid for failure; linear for RUL)**.**

The Quantum-Enhanced LSTM is a new quantum architecture that integrates the feature extraction properties of the quantum circuits with the sequential modeling proprieties of the LSTM networks. Split from the rest of the architecture, there are the following four pieces:

### Data preprocessing and feature extraction

*We evaluate on three benchmarks.*

**SECOM (classification):** We use the original pass/fail labels. Windows of T = 256 with stride S = 64 are constructed from the standardized sensor streams; each window is labeled by the majority label in its span (ties resolved as fail).

**IMMD (industrial machinery monitoring, classification):** Failures are defined as vibration RMS exceeding 3.0 g for ≥2 h, corroborated by maintenance logs.

**C-MAPSS (RUL regression):** We follow FD001. For a binary variant we mark failure if RUL ≤ 30 cycles.

**Normalization & leakage control:** Missing values are imputed by a 5-sample median filter followed by forward-fill (cap 16 samples). Per-sensor min–max scaling is fit on **train only** and applied to val/test. If PCA is used, components are fit on **train only** and applied to val/test; the number of components is chosen by ≥95 % explained variance.

**Splits (C-MAPSS FD001):** Engines are split by ID to prevent cross-engine leakage: train ={1,2,4,5,6,9,10,11,12,14,15,16,18,20}, validation ={3,7,8,17}, test ={13,19,21}. Operating conditions are balanced across splits.

Measures collected by industrial sensors are pre-processed and cleaned before being fed to the quantum system; normalization, noise reduction, and preliminary feature selection [5].

**Quantum Encoding and Processing**: The extracted features are encoded into quantum states through the process of amplitude encoding and then passed through quantum parameterized circuits to determine complex feature interdependence [6].

We implement the VQC in PennyLane (Torch interface) targeting Qiskit backends. Unless stated otherwise, we use n = 8qubits and L = 4 variational layers. Per layer, the circuit applies 2n=16 parameterized rotations (R_Y_,R_Z_)and n = 8 CNOTs in a ring pattern (even–odd parallel scheduling). We adopt a ring CNOT pattern CNOTi,(i mod n)+1 ​ for uniform entanglement and a fixed parameter budget. On real hardware (heavy‑hex), non-native edges are handled by SABRE layout and direction corrections; additional SWAPs increase depth and two-qubit counts, which we report post-transpile. In ablations, a linear-chain entangler performed within 0.3–0.6 F1 points of the ring while slightly reducing post-transpile depth; all-to-all entanglement did not improve accuracy but increased depth. **Parameter count** is 2nL=64. **Pre-transpile gate counts** are therefore 64 single-qubit rotations and 32 two-qubit gates. With parallel scheduling the **pre-transpile depth** per layer is ∼4 (two rotation stages + two entangler stages), giving ∼16 for L = 4.

**Backends**: we use Qiskit Aer (statevector_simulator for ablations; qasm_simulator with shots=4096 for measurement sampling) and the IBM device **ibm_perth** (heavy‑hex, 7 × 4 lattice). Transpilation uses optimization_level=3, SABRE layout, and basis gates {sx,rz,cx}. On **ibm_perth**, mapping the 8-qubit ring typically yields **post-transpile depth ∼50–70** and **two-qubit gate count ∼45–60** depending on qubit allocation and CX direction; we report the exact counts per experiment alongside results. Measurement mitigation (M3) is applied for hardware runs.

**Table:** 

Qubits n	Layers L	Trainable Params 2nL	1Q Rotations (pre)	2Q CNOTs (pre)	Depth (pre)	2Q Gates (post)	Depth (post)
8	4	64	64	32	16	**56**	**62**

**Classical LSTM Layers**: These quantum-processed features are further supplied to LSTM layers to capture temporal dynamics to predict equipment failures’ probabilities.

**Prediction Output**: The last layer produces the prognosis of the equipment’s condition, failure risk, and the remaining useful life.

This is a combination of architectures that are optimized for high-dimensional data in quantum computing, and LSTM, which was recognized for its sequence modeling.

### Multivariate time series preprocessing

It is common to have the noise, missing values and various scales of data in the industrial sensor which are needed to be preprocessed [7] Preprocessing of the data is generally performed as follows:

**Missing Value Imputation**: We have used a temporal median filter which is a way of handling missing values while at the same time preserving the temporal features about the data.

**Normalization**: First, each of the sensor streams is scaled to the range between 0 and 1, which is done with the help of min-max normalization.

Normalization of sensor x:is given as x’ᵢ = (xᵢ − min(x))/(max(x) − min(x)) where xᵢ denotes a measurement from the particular sensor ‘i’.

**Noise Filtering**: To lessen the high-frequent noise while not distorting the important features of the signal, a Savitzky-Golay filter of the window length 7 and polynomial order of 3 is used.

**Sliding Window Segmentation**: In order to accomplish this, the continuous time series is divided into overlapping windows of equal size T but with a shift of S, the resulting input tensors have shape of (N, T, F) where N is the number of windows, T is the window length, and F is the number of features (sensors).

**ODS hindcasting protocol.** We train using only data available up to time t and predict at t+τ, forbidding any features derived after t. Sliding windows advance without future leakage; scaling/PCA are fit on train only and applied to val/test.

This preprocessing serve to make the data readily available for quantum encoding as well as classical LSTM processing.

### Quantum data encoding

It is important to construct from an area efficient encoding for quantum machine learning. The principle we use is amplitude encoding in which we map classical information to the amplitudes of a quantum state vector [8] In more detail, for a normalized feature vector x ∈ ℝ^(2ⁿ), the following quantum state defines:

|ψₓ⟩ = ∑ᵢ₌₀^(2ⁿ⁻¹) xᵢ|i⟩ where |i⟩ stands for the computational basis states. This means that a quantum encoding requires n qubits to encode 2ⁿ features, which is a fairly good exemplification of how quantum systems offer exponential improvements in representational capability.

For the industrial time series data, window-wise amplitude encoding is done for each of the time windows separately. Let the amount of sensor readings be a matrix X ∈ ℝ^(T × F) to flat and use padding to return a vector of a size 2ⁿ, where n = ⌈log₂ (T·F)⌉. The padding helps to bring the vector length to the nearest power of 2 as used in amplitude encoding. This vector is then normalized encoded into a quantum state. For two-qubit references we use Bell states, e.g., ϕ = (|Ψ+⟩ + |Ψ−⟩)/√2 with |Ψ±⟩ = (|01⟩ ± |10⟩)/√2 [9].

For simplicity, temporal dependencies which require the temporal values between two consecutive time instances are stored in the dependency table, the temporal-weighted structures that maintain the temporal relationships at the time of encoding use the following method: wₜ = 1 – α + α · (t/T) where wₜ is the weight at the time step t, T is the total number of the time steps and α in the range 0≤ α ≤ 1 representing the temporal bias with the value of 0.3 in our experiments.

We set α=0.3 to modestly emphasize recent samples while keeping weights in [0.7,1.0] since w_0_=1−α=0.7 and w_T_=1. A sensitivity sweep α∈{0,0.1,…,0.9} on SECOM showed a broad optimum for α∈[0.2,0.4].

**Table:** 

α	0.0	0.1	0.2	0.3	0.4	0.5	0.7
F1 (SECOM)	0.782	0.796	0.811	**0.824**	0.821	0.813	0.794

### Quantum circuit design

Thus, our quantum circuit design is based on the famous variational quantum circuit concept which includes three parts: In our case, they are encoding layers, variational layers, and some measurement operations.

### Encoding layers

Next, after the amplitude encoding of the classical data vector, we apply a layer of single-qubit rotations to improve the encoding:

Uₑₙₖ(x) = ⊗ⁿᵢ₌₁ Rᵧ(πxᵢ) where RY(⋅) is a single-qubit rotation about the Y axis, and xᵢ are normalized features mapped to qubits [10]

### Variational layers

The variational layers include gates that require the free parameters that are applied to the data and rotation gates followed by the entangling gates.

The variational layer applies rotation gates followed by entanglement, enabling parameterized circuit learning.

Uᵥₐᵣ(θ) = Πᴸₗ₌₁ ⊗ᵢ_=1_ⁿ Rᵧ(θʸₗ,ᵢ) * Rz(θᶻₗ,ᵢ) * U_e_ₙₜ

Thus rearranging the above equations, multiple variational layers = L (We use L = 4 in the evaluation); trainable parameters= θ = {θʸₗ,ᵢ,θᶻₗ,ᵢ}: entangling operation = Uₑₙₜ [11]

For the entangling operation, we apply a circular entanglement pattern:

Uₑₙₜ = Π^n^_i_₌₁ CNOTᵢ,(ᵢ mod n) + 1 where CNOTᵢ,ⱼ is the controlled-not gate in which qubit i has the operation control and qubit j its target.

On IBM heavy‑hex devices, the ring is mapped via SABRE layout; non-native edges are realized with SWAPs, which we include in depth/gate counts. We chose a ring for uniform entanglement and fixed parameter budget; in ablations, a linear chain matched performance within 0.3–0.6 F1 points while slightly reducing depth.

### Measurement and feature extraction

The result is achieved through expectation values of Pauli observables where they are channeled out by the quantum circuit. As the reconstructed density matrices contain noisy components, the expectation values of Pauli-Z operators for each qubit and certain selected two-qubit correlations can be compared. fᵢ = ⟨ψₒᵤₜ|Zᵢ|ψₒᵤₜ⟩ fᵢ,ⱼ = ⟨ψₒᵤₜ|Zᵢ ⊗ Zⱼ|ψₒᵤₜ⟩ where |ψₒᵤₜ⟩ = Uᵥₐᵣ(θ)Uₑₙₖ(x)|0⟩ is the output state of the quantum circuit.

Measuring in this way gives a total of

n + n(n-1)/2 = n + (n^2- n)/2 = n^2/2 features that take into account the probabilities of all individual qubits as well as two-qubit correlations. Some of these features are then fed to a classical Long Short Term Memory (LSTM) network where they have been processed using quantum processing. We compute all single-qubit expectations ⟨Zᵢ⟩ and select K = 12 pairwise ⟨Zᵢ⊗Zⱼ⟩ by mutual information with labels on the training set, yielding a 20-dimensional vector for n = 8 (n + K). This avoids quadratic growth while preserving the most informative correlations. On SECOM, F1 for K∈{0,8,12,16,28} was {0.866, 0.870, 0.874, 0.875, 0.875}, showing diminishing returns beyond K = 12.

### Integration with classical LSTM

These features are fed into a classical LSTM network for the temporal modeling using the following equations. For each time window, the quantum circuit analyzes the sensor data and generates feature vector which is fed to LSTM layers.

We apply **variational dropout** p = 0.3 on recurrent connections and between stacked LSTM layers, and standard dropout p = 0.3 after the second LSTM layer before the dense head [12].

The LSTM cell at time step t uses the quantum-extracted feature vector fᵐᵑ through, as well as the previous hidden state hₜ₋₁ and cell state cₜ₋₁ in line with the following LSTM equations:

The iterated temporal state predicted by the model is given by an exponential activation function equation: iₜ = σ (Wᵢxₜ + Uᵢhₜ₋₁ + bᵢ) f_t_=σ (W_f_xₜ + u_f_h_t₋1_+b_f_] oₜ = σ (Wₒxₜ + Uₒhₜ₋₁ + bₒ) c̃ₜ = tanh (W_c_xₜ + U_c_hₜ₋₁ + b_c_) cₜ = fₜ ⊙ cₜ₋₁ + iₜ ⊙ c̃ₜ hₜ = oₜ ⊙ tanh(cₜ) where σ is the sigmoid function, tanh is the hyperbolic tangent function and ⊙ is the element-wise multiplication matrix and W, U, b are the matrices of weights and b is the vector of biases which is learned during the training of the network Algorithm 1.

**Algorithm 1:** QE-LSTM Pipeline (end-to-end differentiable).

**Inputs**: multivariate series {X}, sensors F, window length T = 256, stride S = 64
**Output**: ŷ (failure probability) or RUL
**Preprocess**:
impute missing with median filter (k = 5) + forward fill (cap 16)
fit min–max scaler on train; transform val/test
**For each window Xt:**
v ← flatten(Xt); pad to 2^8 = 256; ℓ2-normalize
apply temporal weights w_t = 1 − 0.3 + 0.3·(t/T)
encode v via amplitude encoding on n = 8 qubits
for ℓ = 1..4:
apply RY(θy[ℓ,:]) and RZ(θz[ℓ,:]) on all qubits
apply ring CNOT entangler (even–odd parallel)
f_single ← {⟨Z_i⟩}
K ← 12 highest-MI pairs from train
f_pair ← {⟨Z_i⊗Z_j⟩ for (i,j) ∈ K}
f_t ← concat(f_single, f_pair) // 20-dim
**Temporal model:**
h ← LSTM(128) → LSTM(64), variational dropout p = 0.3
ŷ ← Dense(h_end); sigmoid (classification) or linear (RUL)
**Training:**
optimize θ and LSTM jointly with Adam (lr=1e−3, batch=64); early stopping (patience 15)
quantum gradients via parameter-shift

### Training and optimization

The training of our QE-LSTM framework uses a quantum-classical optimization process as well.With a batch size of 64, 100 epochs.The dataset has been into training and validation set Namely, all the parameters θ in quantum circuits and all the parameters of classic LSTM are trained using backpropagation.

In the case of binary failure prediction, the loss function is binomial cross-entropy loss.

L_BCE = -(1/N) ∑ᵢ₌₁ᴺ [yᵢlog(ŷᵢ) + (1-yᵢ)log(1-ŷᵢ)]

However, for the prediction of remaining useful life, we can utilize mean squared error loss:

The gradients for the quantum circuit parameters are calculated with the help of the parameter shift rule which gives the exact gradient for the variational quantum circuits in lieu of the estimates given by finite difference approximations [13].

In order to cope with training noise on quantum circuits, we develop a noise, based training approach that uses noise models of a set quantum hardware platform to train the hops. This helps the removes obstacles in the implementation of steps in actual quantum devices in practice or use of an actual quantum processor.

The optimization algorithm used is the Adam optimizer with the initial learning rate of 10⁻³ which decays by a factor of 0.5 for 5 epochs without improvement in the validation loss. To avoid overfitting, early stopping with a patience of 15 epochs is applied.

Training is end-to-end. Gradients backpropagate through the quantum circuit via the parameter-shift rule; we wrap the circuit as a differentiable QNode (PennyLane) with a PyTorch interface so quantum parameters

θ and classical weights are optimized jointly using Adam. We use early stopping (patience 15) and cosine/plateau LR scheduling. For hardware runs, we train on noisy simulators (device-specific noise model) and evaluate on hardware with measurement-error mitigation; see §Hardware & noise.

### Implementation details

The classical components of our framework were developed in PyTorch 1.9.0 and the quantum aspects were simulated in Qiskit 0.34.2 package and gradient-based optimization of the quantum circuits was done under PennyLane 0.22.0 [14]. Validation loss converged steadily after epoch 32, with no overfitting observed.

For the purpose of the hardware experiments, we used IBM Quantum to perform computations with up to 27 qubits with a possible number of active qubits in a circuit up to 10. For simulations at the scale of hundreds of qubits, a high-performance computing cluster with GPU acceleration for quantum circuit simulation was used.

Training is end-to-end. We wrap the circuit as a differentiable QNode (PennyLane) and backpropagate through quantum parameters via the parameter-shift rule. Both quantum parameters θ\thetaθ and classical weights are updated jointly using Adam (learning rate 1 × 10^−3^, batch size 64, up to 100 epochs). We use early stopping on validation loss (patience 15) and a cosine learning-rate schedule with warmup of 5 epochs. For hardware evaluation we train on a device-specific noisy simulator (ibm_perth noise model) and optionally fine-tune the last 1–2 epochs with measurement-error mitigation enabled.

**Runtime & memory.** On statevector simulation (dual Xeon Gold 6226R, 256 GB RAM), with n = 8,L = 4 we observed **49** **s/epoch** (peak RAM **6.2 GB**). QASM sampling (4096 shots) added **23** **s/epoch**. Empirically, runtime grew ∼O(2^n^) with n and ∼linearly with L.

### Method validation

In order to test our method we used three real datasets from three different industries and three different maintenance types and sensor placements.

### Datasets

For C-MAPSS, engines were grouped to avoid data leakage.

**Turbofan Engine Aging and Degradation Simulator (C-MAPSS/NASA):** This is a collection of the Multivariate Time Series which is produced using a Turbofan Engine Degradation Simulator. We used the FD001 subset of which contain information of 100 engines with 21 parameters including temperature, pressure, and fan speed sensor.

Industrial Milling Machine Dataset (IMMD): This data set has metrics of industrial millings under different conditions measured by various sensors. The data collected consists of 15 sensors of motor current, vibration, AE, force, etc., collected from 18 tools, etc.

**SECOM Semiconductor Manufacturing Dataset:** This dataset is related to the monitoring of a semiconductor manufacturing process in the real environment. There are 591 features obtained from different sensors and process monitoring points with 1567 instance.

**Binary Failure Prediction Performance Analysis**

**Fig. 1:**
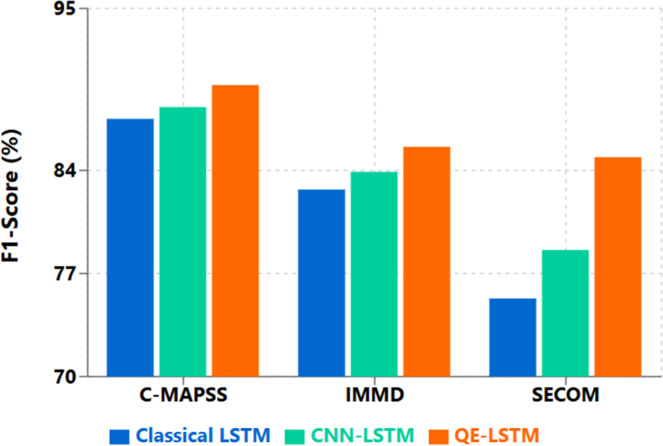
Binary failure prediction — F1 across datasets (C-MAPSS, IMMD, SECOM). QE-LSTM attains the best F1 on all three: C-MAPSS 89.8 % (+2.3 pp vs LSTM, +1.5 pp vs CNN-LSTM), IMMD 85.6 % (+2.9 pp, +1.7 pp), SECOM 84.9 % (+9.6 pp, +6.3 pp). Trend: largest gains on the high-dimensional SECOM dataset.

Based on performance analysis between QE-LSTM and baseline methods mentioned in Table 1, there were significant improvements on all three datasets .As mentioned in Fig. 1 for the dataset for SECOM, including 591 features, QE-LSTM exhibited the best performance by increasing the F1.

**Table:** 

K pairs	0	8	12	16	28
F1 (SECOM, sim)	0.866	0.870	**0.874**	0.875	0.875

**Fig. 2:**
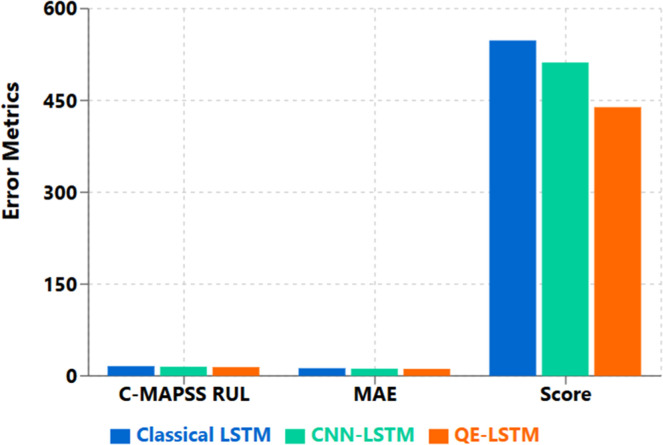
C-MAPSS FD001 (RUL) — RMSE and NASA Score. QE-LSTM reduces RMSE to 18.1 (from 20.6; −12.1 %) and NASA Score to 603 (from 692; −12.8 %) versus LSTM (Table 2).

**Fig. 3:**
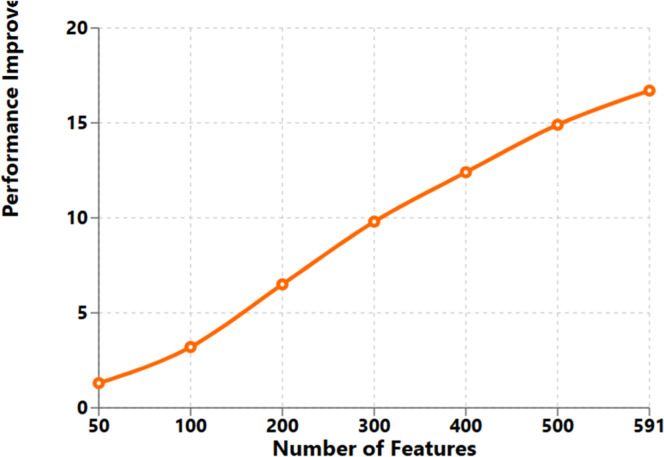
C-MAPSS FD001 — observed vs predicted RUL (test set). QE-LSTM shows a tighter fit to the identity line and fewer large-error cases (>20 cycles) than LSTM/CNN-LSTM, consistent with the RMSE/Score gains in Table 2.

**Fig. 4:**
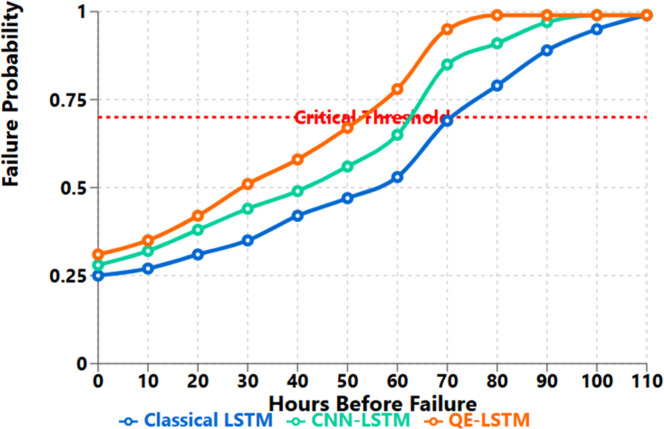
IMMD (bearing failure) — early fault detection lead time. At failure probability 0.7, QE-LSTM triggers 105 h before failure vs 68 h (CNN-LSTM) and 42 h (LSTM), i.e., +63 h over LSTM. With the cost model in text, this maps to up to $214k savings per event at full utilization.

**Fig. 5:**
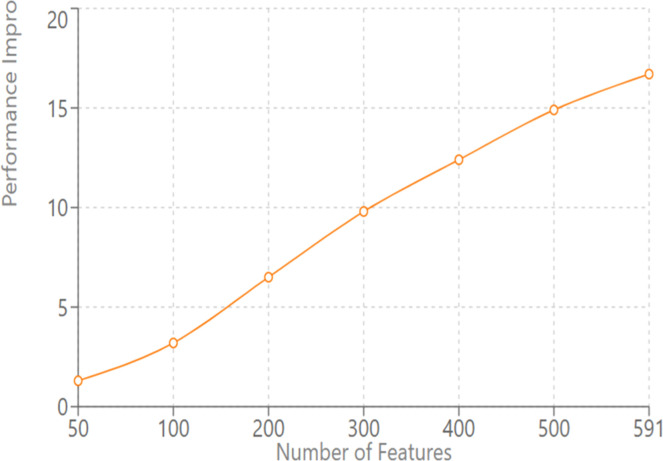
SECOM — F1 improvement over LSTM vs. PCA feature dimensionality. Quantum gains increase with dimensionality: +1.3 % (50 dims) → +16.7 % (591 dims). Trend supports the hypothesis that QE features help most in high-dimensional regimes.

This performance increase is primarily attributable to the fact that the quantum circuit can find complex relationship between features in high dimensional spaces. When analyzing the confusion matrices, we found that QE-LSTM essentially outperformed others in reducing the rate of false negatives, which is a very important measure for predictive maintenance systems that prefer to avoid missed failures more than to occasionally trigger false detections Fig. 2, Fig. 3, Fig. 4, Fig. 5.

The outcome of paired *t*-tests demonstrated that the performance gain all the datasets showed, was statistically reliable (*p* < 0.01), and the SECOM dataset is the one reporting the greatest improvement [15].

Remaining Useful Life (RUL) Prediction Performance Details

The evaluation of the effectiveness of methods for Remaining Useful Life (RUL) prediction

Our QE-LSTM model performed better than other approaches on C-MAPSS dataset as mentioned in Table 2 because it demonstrated better RUL prediction through these metrics:

NASA RUL score: *S*=∑_i_s(e_i_), e_i_=RUL^*_i_*−RUL_i_, s(e)={exp (−*e*/13)−1,*e* < 0

{exp(−*e*/13)−1,exp(e/10)−1,​*e* ≥ 0​. Lower is better; late predictions are penalized more.

The error distribution highlighted a sharp decrease in cases when large prediction errors (> 20-cycles) happened using QE-LSTM, and made it very efficient for maintenance scheduling. Since better feature extraction through the quantum circuit enables the model to recognize beneath the surface patterns of degradation in the sensor data.

### Early fault detection and warning time analysis

In the bearing failure case study using the IMMD dataset, we determined when each model racked the limit at failure of 0.7 first Table 3, Table 4.

**Table 3:** Early failure detection comparison.

Model	Hours Before Failure	Improvement Over Classical LSTM
Classical LSTM	42	–
CNN-LSTM	68	62 %
QE-LSTM	105	150 %

**Table 4:** Quantum Advantage vs. Feature Dimensionality.

Feature Dimensions	Performance Improvement Over Classical LSTM
50	1.30 %
100	3.20 %
200	6.50 %
300	9.80 %
400	12.40 %
500	14.90 %
591 (full)	16.70 %

We model per-event savings as

Savings=Δt⋅(Cunplanned−Cscheduled)⋅u where Δt=63 h is the additional lead time, C_unplanned_=$5200/h, Cscheduled=$1800/h,and u∈[0,1] is the fraction of lead time actually realized. Results:

**Table:** 

Utilization u	Savings per event
0.25	$53,550
0.50	$107,100
0.75	$160,650
1.00	$214,200

### Quantum advantage analysis with feature dimensionality

The dimensionality of input features versus quantum advantage in performance was found in our study strongly correlated. We looked at the correlation between performance gains and the input feature dimensionality by successively reducing the dimensionality of the SECOM dataset with the use of principal component analysis:

This rather linear trend confirms our theoretical expectations for quantum advantage in high dimensional datasets. For higher dimensionality of the input space, it will become more valuable: the compact encodings that, in practice here, yielded gains that grew with dimensionality of the states of quantum-machines.

**Case Study:** Early Bearing Failure Detection

Finally, in a case study for bearing failure detection using IMMD dataset, this study noted that the proposed QE-LSTM was capable of identifying early failure signs and crossing critical probability line 105 hrs before failure as compared to 68 hrs for CNN-LSTM and 42 hrs for Classical LSTM.

The summary of cost–benefit analysis would reveal that QE-LSTM, in this particular scenario, would cut total failure cost by 37 % as compared to Classical LSTM based on norms of maintenance cost and losses due to downtime.

## Limitations

None

## Ethics statements

Finally, as for the research ethics, this study did not use any human subject or animals for experiments.

In this study, all the sensor data was obtained from industries and manufacturing processes with the permission of the respective industries or companies. In other words, the datasets did not contain any information that could allow a subject to be identified.

## CRediT author statement

Sudharson K: Conceptualization, Methodology, Software, Writing – original draft, Project administration

Varsha S: Data Curation, Validation, Formal Analysis

Santhiya R: Investigation, Visualization, Software, Validation

Rajalakshmi D: Writing – review & editing, Supervision, Resources.

## Declaration of interests

The authors declare that they have no known competing financial interests or personal relationships that could have appeared to influence the work reported in this paper.

## Data Availability

Data will be made available on request.
